# Understanding knowledge, perception, and willingness of non-invasive prenatal testing for fetal aneuploidy: a survey among Chinese high-risk pregnant women

**DOI:** 10.3389/fmed.2023.1232942

**Published:** 2023-10-16

**Authors:** Yi Zhao, Zhu Xue, Yarui Geng, Jie Zhu, Maidan Hu, Minmin Jiang

**Affiliations:** ^1^Department of Drug Clinical Trials, Women’s Hospital, School of Medicine, Zhejiang University, Hangzhou, China; ^2^Shulan International Medical College, Zhejiang Shuren University, Hangzhou, China

**Keywords:** non-invasive prenatal testing, cell free DNA, prenatal screening, genetic counseling, aneuploidy, pregnant women

## Abstract

**Objectives:**

Non-invasive prenatal testing (NIPT) is utilized for screening the likelihood of fetal aneuploidy, presenting the benefits of non-invasiveness, high sensitivity, and specificity. Its application in prenatal screening has become ubiquitous. The inquiry into how pregnant women comprehend and determine NIPT screening strategies is paramount. Regrettably, there has been a dearth of research on this subject in China. Consequently, this study scrutinizes pregnant women’s cognizance and perspectives concerning NIPT, furnishing a foundation for advancing its judicious implementation.

**Methods:**

From February 2021 to December 2022, a questionnaire survey was conducted among pregnant women receiving prenatal care and screening at the Women’s Hospital, School of Medicine, Zhejiang University, who were randomly selected from a pool of individuals exhibiting a high risk of fetal aneuploidy on serological screening. The survey aimed to gather data on participant characteristics, knowledge, perception, and willingness concerning NIPT. The study employed chi-square and Kruskal Wallis tests to analyze subgroup differences.

**Results:**

A total of 226 valid questionnaires were obtained. 83.2% of women pregnant women identified as high risk by serological screening would opt for NIPT, with 66.4% indicating that they would prefer NIPT for fetal aneuploidy screening in future pregnancies. These findings suggest a notable willingness among pregnant women to undergo NIPT. Additionally, the results suggest that various factors, including place of residence, educational level, family income, causes of abortion, and conception method, influence pregnant women’s knowledge about NIPT Accordingly, the level of NIPT knowledge varies among pregnant women.

**Conclusion:**

The survey generally revealed that pregnant women were strongly inclined to select NIPT; however, expectant Chinese mothers possess limited knowledge and perception regarding this screening method for fetal aneuploidy. Therefore, the government must implement effective measures to augment public awareness of fetal aneuploidy screening and encourage the judicious utilization of NIPT.

## Introduction

Non-invasive prenatal testing (NIPT), also referred to as non-invasive prenatal screening (NIPS), refers to the screening of free placenta-derived DNA in maternal blood serum and is a non-invasive method for detecting fetal aneuploidy (1, 2). Unlike invasive prenatal diagnosis, NIPT minimized the risk such as miscarriage or infection. Since its introduction in Hong Kong in 2011, NIPT has been adopted in over 60 countries due to its high sensitivity (3, 4) and specificity in identifying fetal chromosomal abnormalities, particularly trisomies 21, 18, and 13 (5). The American College of Medical Genetics and Genomics (ACMG) advocates for NIPT as the primary method for detecting fetal aneuploidy (6). In contrast, many other countries, including China (7) and the United Kingdom (8), consider serological screening to be the primary approach, using NIPT as a secondary screening method for high-risk pregnancies (1, 4, 5). NIPT is not utilized as the initial screening method due to its ability to detect more chromosomal abnormalities, which would result in increased expenses. Cost-effectiveness analyses indicate that utilizing NIPT as a secondary screening method following traditional prenatal scanning methods is more economically viable (5).

In 2016, the China National Health and Family Planning Commission promulgated “the technical specifications of Prenatal Screening and Diagnosis of Fetal Free DNA in Peripheral Blood of Pregnant Women,” which delineated criteria for the optimal timing, eligible and ineligible candidates for NIPT (9). The guidelines stipulated that serological prenatal screening is the primary method for detecting fetal aneuploidy, whereas NIPT is a secondary screening modality (9). Simultaneously, Chinese technical experts have achieved a consensus regarding the laboratory process of prenatal screening utilizing maternal peripheral blood fetal free DNA (10) and experts’ quality evaluation indicators for prenatal screening (11).

The Chinese government and experts attribute substantial importance to NIPT (12), manifesting in the publication of its Technical Specifications (7) and consensus on quality assessment indices (11). However, it is critical that pregnant women, being the subjects of NIPT application, comprehend the screening plan meticulously, as it forms the underpinning of its effective implementation. Documentary research revealed that scholars from multiple nations, including the United States (13), Italian (14), Croatian (15), Canada (16), Australia (17), and Hong Kong, China (18), have conducted studies on the knowledge and willingness of pregnant women regarding NIPT for fetal aneuploidy. However, heretofore, investigations regarding the awareness and inclination of pregnant women in Mainland China have not been reported. Therefore, in light of this, the present study delves into pregnant women’s cognition and standpoints pertaining to NIPT in Mainland China, thereby providing a solid foundation for facilitating its prudent utilization.

## Materials and methods

### Study design

The investigators created a survey instrument that drew upon insights from prior research (17, 18) and was organized into two distinct parts. The initial section of the questionnaire was designed to gather demographic data from the study participants. In contrast, the second section focused on eliciting their evaluations of knowledge, perception, and willingness concerning NIPT screening. The Knowledge, Perception, and Willingness Questionnaire (KPQ) comprised a total of 17 items, including 10 knowledge-based queries (K, K1–K10) (19), five perception-based queries (P, P1–P5), and two willingness-based queries (W). The survey was initially developed in English and subsequently translated into Chinese by two bilingual Chinese speakers with a strong command of English. The study was approved by the Women’s Hospital, School of Medicine, Zhejiang University Ethics Committee (approval number: IRB-20210185-R), and obtained informed consent from each participant following the Helsinki Declaration.

### Participants and data collection

The study was conducted at Women’s Hospital, School of Medicine, Zhejiang University, from February 2021 to December 2022. The inclusion criteria for the study were pregnant women between 12^+0^ and 22^+6^ weeks gestation who had undergone Pregnancy Health Record and prenatal screening at Women’s Hospital, School of Medicine, Zhejiang University, were proficient in reading and speaking Chinese, provided informed consent, and exhibited a critical risk of fetal common chromosomal aneuploidy based on serum screening results between the high-risk cutoff value and 1/1,000. The exclusion criteria included pregnant women with a history of mental illness, medical students, resident physicians, and researchers, women who had discussed NIPT with a clinical doctor previously, gestational age less than 12^+0^ weeks, couples with a known chromosomal abnormality, those with who had received an allogeneic blood transfusion, transplantation surgery, or cell therapy within the past year, fetal ultrasound examination suggesting structural abnormalities requiring further prenatal diagnosis, those with a family history of genetic diseases or a high risk of fetal genetic disease, and those with malignancies during pregnancy. Additionally, the participants were not compensated for participating in the study.

The raw data collected were securely stored in an Excel spreadsheet. When alerted of an critical risk for fetal common chromosomal aneuploidy stemming from serum screening outcomes in pregnant women, researchers promptly commenced telephonic or face-to-face conversations and consultations to determine their preliminary inclination towards participation in this study. Before responding to the survey, participants were provided with a concise overview of the research, encompassing its objectives, anonymity assurance, methodology, and guidelines for completing the questionnaire. The study adhered to all privacy regulations and refrained from including any extraneous inquiries in the survey.

### Sample size and sampling procedure

As per the previous observation (20, 21), it is recommended that the sample size for the early questionnaire should be a minimum of four times the number of questionnaire items. Recent research has also suggested a minimum sample size based on a subject with a variable ratio of 5:1 (22, 23). With the inclusion of 17 items in this questionnaire and assuming no response bias, selecting “I do not know/I am not sure” from Part B could require a 35% increase in the sample size. Consequently, the ideal sample size should be 5 × 17 × (1 + 35%) × 2 = 230.

### Statistical analysis

Data were analyzed by the SPSS statistical software (version SPSS 20.0, IBM SPSS Inc., Chicago, IL, United States). Count data are expressed as percentages (%), while measurement data are presented as mean ± SD, and the chi-square test or Kruskal Wallis test was used as appropriate. The significance Kruskal-Wallis test level alpha was set at 0.05, and *p* ≤ 0.05 indicated statistically significant differences.

## Results

### Basic characteristics of the participants

Between February 2021 and December 2022, 235 questionnaires were disseminated to pregnant women who were enrolled for prenatal care and screening at the Women’s Hospital, School of Medicine, Zhejiang University, and who fulfilled the inclusion criteria. Of these, 231 (98.3%) were retrieved, and 226 (96.2%) were deemed valid. Among the 226 participants, 37.6% were aged 35 years or older, 72.6% resided in urban areas, 69.0% held a bachelor’s or college degree, 56.2% had no prior childbirth experience, 43.8% were primipara or multipara, 58.4% had a history of previous miscarriage or termination of pregnancy, and 27.4% had experienced fetal defects, 73.9% conceived naturally, and 91.6% were in gestational weeks 12^+0^–22^+6^. The basic characteristics of the participants are shown in Table 1.

**Table 1:** Basic characteristics of the participants (*n* = 226).

Characteristics	*N* (%)
Age	
<35 years	141 (62.4)
≥35 years	85 (37.6)
Place of residence	
Urban	164 (72.6)
Rural	62 (27.4)
Educational level	
Less than high school	47 (20.8)
Bachelor/College degree	156 (69.0)
Master or above	23 (10.2)
Family income (per month)	
<5,000(RMB)	20 (8.8)
5,000–10,000(RMB)	70 (31.0)
≥10,000(RMB)	136 (60.2)
Parity	
0	127 (56.2)
≥1	99 (43.8)
Previous miscarriage or termination of pregnancy	
Yes	132 (58.4)
No	94 (41.6)
Causes of abortion^a^	
Fetal defects	62 (27.4)
Non-fetal defects	70 (31.0)
Conception way	
Natural conception	167 (73.9)
Assisted reproduction technology	59 (26.1)
Gestational weeks (week)	
12^+0^–22^+6^	207 (91.6)
23^+0^–30^+6^	19 (8.4)

All chi-square tests, educational level and family income except for the Kruskal-Wallis test.

a 132 participants had previous miscarriages or termination of pregnancy.

### Pregnant women’s knowledge of NIPT

Table 2 elucidates respondents’ cognizance concerning NIPT, with Knowledge Questions 1–5 broaching foundational aspects related to NIPT, while Questions 6–10 delve into intricate issues about NIPT’s relevance to atypical risk groups. From the study, it was gleaned that 60.6% of pregnant women understand that NIPT is only a screening test for detecting chromosomal abnormalities and that a diagnostic test is required through invasive procedures (K1 in Table 2). Additionally, 76.5% recognize that NIPT results may not be informative (K2 in Table 2). The understanding that NIPT fails to pinpoint all instances of fetal Down syndrome was clear to 65% of the female respondents, while an impressive 72.6% discerned that occasional false-positive results compel those with positive NIPT outcomes to pursue verification via auxiliary tests such as chorionic villus sampling or amniocentesis (K3 and K4 in Table 2). Concurrently, 90.7% of pregnant women maintained that a positive marker in the NIPT would precipitate elevated stress levels (K5 in Table 2). However, regarding the knowledge of NIPT’s applicability to special risk groups (K6–K10 in Table 2), correct answers in the other questions did not exceed 50%, except for K7 (54.4%).

**Table 2:** Responses of KPW questions on non-invasive prenatal testing (NIPT) (*n* = 226).

Questions	Yes; *n* (%)	No; *n* (%)	Do not know; *n* (%)
K1: NIPT can detect only half of the fetal chromosomal abnormalities that would be identified through amniocentesis or chorionic villus sampling	137 (60.6)^a^	21 (9.3)	68 (30.1)
K2: For some women, a NIPT test result may not be informative because of an inadequate amount of fetal DNA in maternal plasma or other reasons	173 (76.5)^a^	11 (4.9)	42 (18.6)
K3: NIPT does not detect all cases of fetal Down syndrome	147 (65.0)^a^	19 (8.4)	60 (26.5)
K4: There are also occasional false-positive results, and therefore women with positive NIPT results need to receive confirmatory testing through chorionic villus sampling or an amniocentesis	164 (72.6)^a^	14 (6.2)	48 (21.2)
K5: Women with positive NIPT results are at very high risk of Down syndrome, and for some women, the extended period awaiting confirmatory invasive testing results is likely to be highly stressful	205 (90.7)^a^	4 (1.8)	17 (7.5)
K6: NIPT is suitable for those women who have or with a family history of a chromosomal abnormality carrying an increased risk of inheritance to their child	113 (50.0)	28 (12.4)^a^	85 (37.6)
K7: NIPT is suitable for multiple pregnancies	53 (23.5)	123 (54.4)^a^	50 (22.1)
K8: NIPT is suitable for pregnancies conceived after a donor *in vitro* fertilization	86 (38.1)	90 (39.8)^a^	50 (22.1)
K9: NIPT is suitable for detecting fetal single-gene disorders such as thalassemia	104 (46.0)	48 (21.2)^a^	74 (32.7)
K10: NIPT is suitable for those women who recently received a blood transfusion, organ transplant, or stem cell therapy	68 (30.1)	70 (31.0)^a^	88 (38.9)
P1: Do you believe that prenatal screening and diagnosis are the primary measures for decreasing the incidence of birth defects?	196 (86.7)	3 (1.3)	27 (11.9)
P2: Do you believe all pregnant women should undergo prenatal screening for chromosomal diseases?	118 (52.2)	19 (8.4)	89 (39.4)
P3: Do you believe that the government should include NIPT in the healthcare insurance system?	194 (85.8)	10 (4.4)	22 (9.7)
P4: Do you believe NIPT is more accurate and diagnostic-like than serum screening for chromosomal diseases?	117 (51.8)	22 (9.7)	87 (38.5)
P5: Do you believe that a medical professional’s recommendations could significantly impact your decision regarding the selection of chromosomal disease screening methods?	216 (95.5)	10 (4.4)	0 (0)
W1: Would you choose NIPT to detect fetal aneuploidy if your serum screening results indicate a high risk of chromosomal abnormalities in your next pregnancy?	188 (83.2)	8 (3.5)	30 (13.3)
W2: Would you opt for NIPT as a primary method for fetal aneuploidy screening in your next pregnancy?	150 (66.4)	26 (11.5)	50 (22.1)

K represents knowledge-based questions, P represents perception-based questions, and W represents willingness-related questions. NIPT represents non-invasive prenatal testing.

a Correct answers were marked.

**Figure 1:**
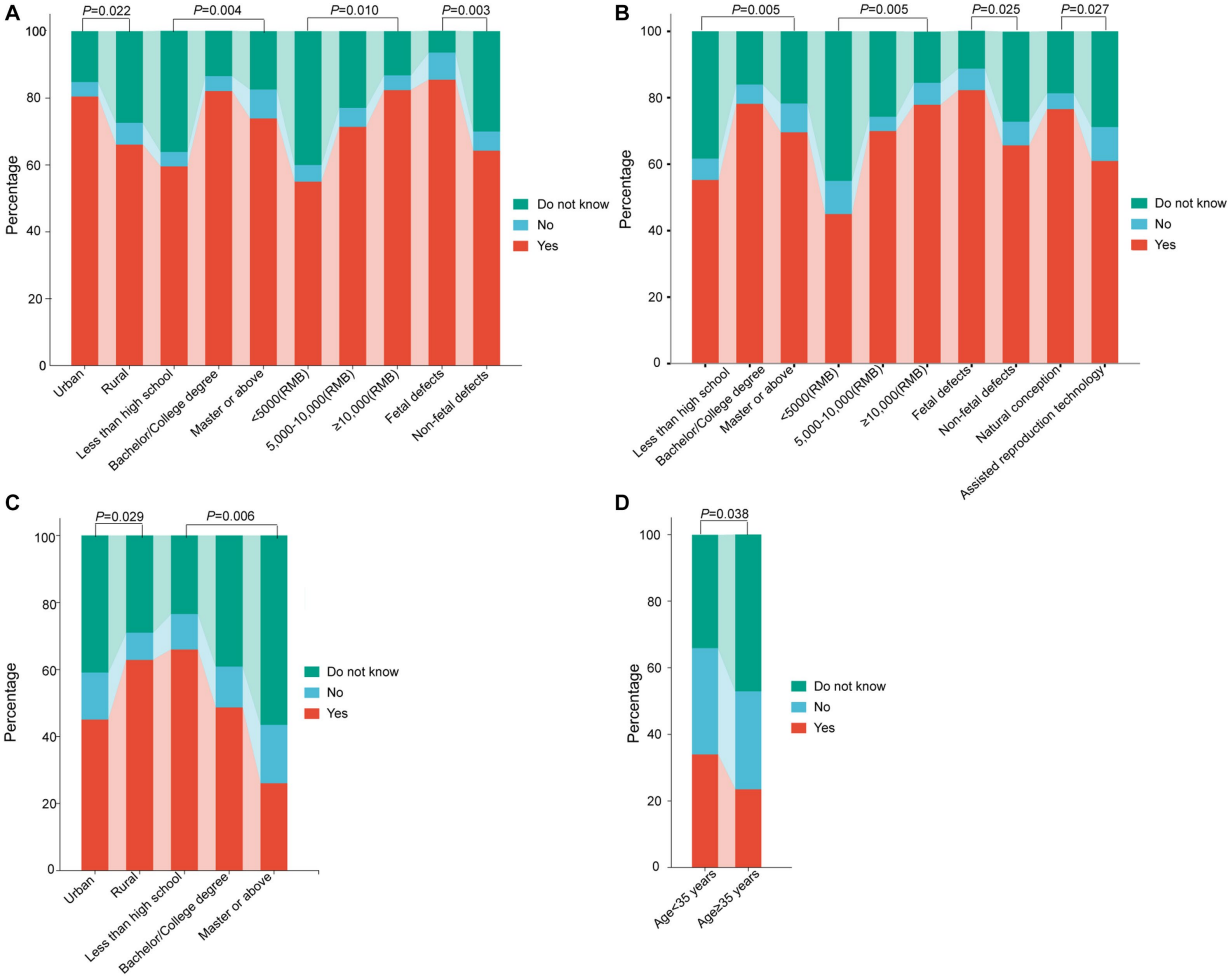
Comparison of pregnant women’s knowledge of NIPT test results among different subgroups. **(A)** Knowledge-based questions 2: For some women, a NIPT test result may not be informative because of inadequate amount of fetal DNA in maternal plasma or other reasons. **(B)** Knowledge-based questions 4: There are also occasional false-positive results, and therefore women with positive NIPT results need to receive confirmatory testing through chorionic villus sampling or an amniocentesis. **(C)** Knowledge-based questions 6: NIPT is suitable for those women who have or with a family history of a chromosomal abnormality carrying an increased risk of inheritance to their child. **(D)** Knowledge-based questions 10: NIPT is suitable for those women who recently received a blood transfusion, organ transplant, or stem cell therapy. NIPT represents non-invasive prenatal testing.

Subsequently, we next compared pregnant women’s knowledge of NIPT test results among different subgroups. Regarding the K2, significant differences were observed in the understanding of NIPT test results not being informative among pregnant women with different places of residence, educational levels, family incomes, and causes of abortion (*p* = 0.022; 0.004; 0.010; 0.003) (Table 3, Figure 1A). With regards to the K4, notable disparities surfaced among expectant women with divergent educational backgrounds, familial income levels, reasons for termination of pregnancy, and modes of conception (*p* = 0.005, 0.005, 0.025, 0.027) (Table 3, Figure 1B). Concerning the K6, pregnant women residing in rural areas or lower education levels had significantly lower correct response rates than their counterparts living in urban areas or higher education (*p* = 0.029, 0.006) (Table 3, Figure 1C). Pertaining to the K10, the incidence of correct responses was markedly elevated in the advanced maternal age (AMA) group (≥35 years) as compared to the non-AMA group (*p* = 0.038) (Table 3, Figure 1D).

**Table 3:** Comparison of pregnant women’s knowledge of NIPT test results among different subgroups.

Characteristics		K1	K2	K3	K4	K5	K6	K7	K8	K9	K10
Age	<35 years	0.523	0.145	0.918	0.464	0.909	0.615	0.855	0.119	0.951	0.038
	≥35 years										
Place of residence	Urban	0.120	0.022	0.360	0.260	0.485	0.029	0.636	0.545	0.111	0.142
	Rural										
Educational level	Less than high school	0.103	0.004	0.058	0.005	0.210	0.006	0.431	0.155	0.135	0.855
	Bachelor/College degree										
	Master or above										
Family income (per month)	<5,000 (RMB)	0.151	0.010	0.124	0.005	0.105	0.144	0.828	0.124	0.975	0.669
	5,000–10,000 (RMB)										
	≥10,000 (RMB)										
Parity	0	0.407	0.168	0.175	0.146	0.154	0.165	0.212	0.544	0.740	0.476
	≥1										
Previous miscarriage or termination of pregnancy	Yes	0.992	0.413	0.781	0.651	0.590	0.576	0.652	0.239	0.915	0.951
	No										
Causes of abortion^a^	Fetal defects	0.083	0.003	0.996	0.025	0.443	0.232	0.194	0.586	0.052	0.432
	Non-fetal defects										
Conception way	Natural conception	0.463	0.105	0.902	0.027	0.478	0.766	0.129	0.599	0.986	0.933
	Assisted reproduction technology										
Gestational weeks (week)	12^+0^–22^+6^	0.528	0.186	0.095	0.238	0.884	0.280	0.927	0.712	0.849	0.734
	23^+0^–30^+6^										

K represents knowledge-based questions; values in the tables were expressed as values of *p*; all chi-square tests, educational level, and family income except for the Kruskal-Wallis test.

a 132 participants had previous miscarriages or termination of pregnancy.

### Pregnant women’s perception of NIPT

**Figure 2:**
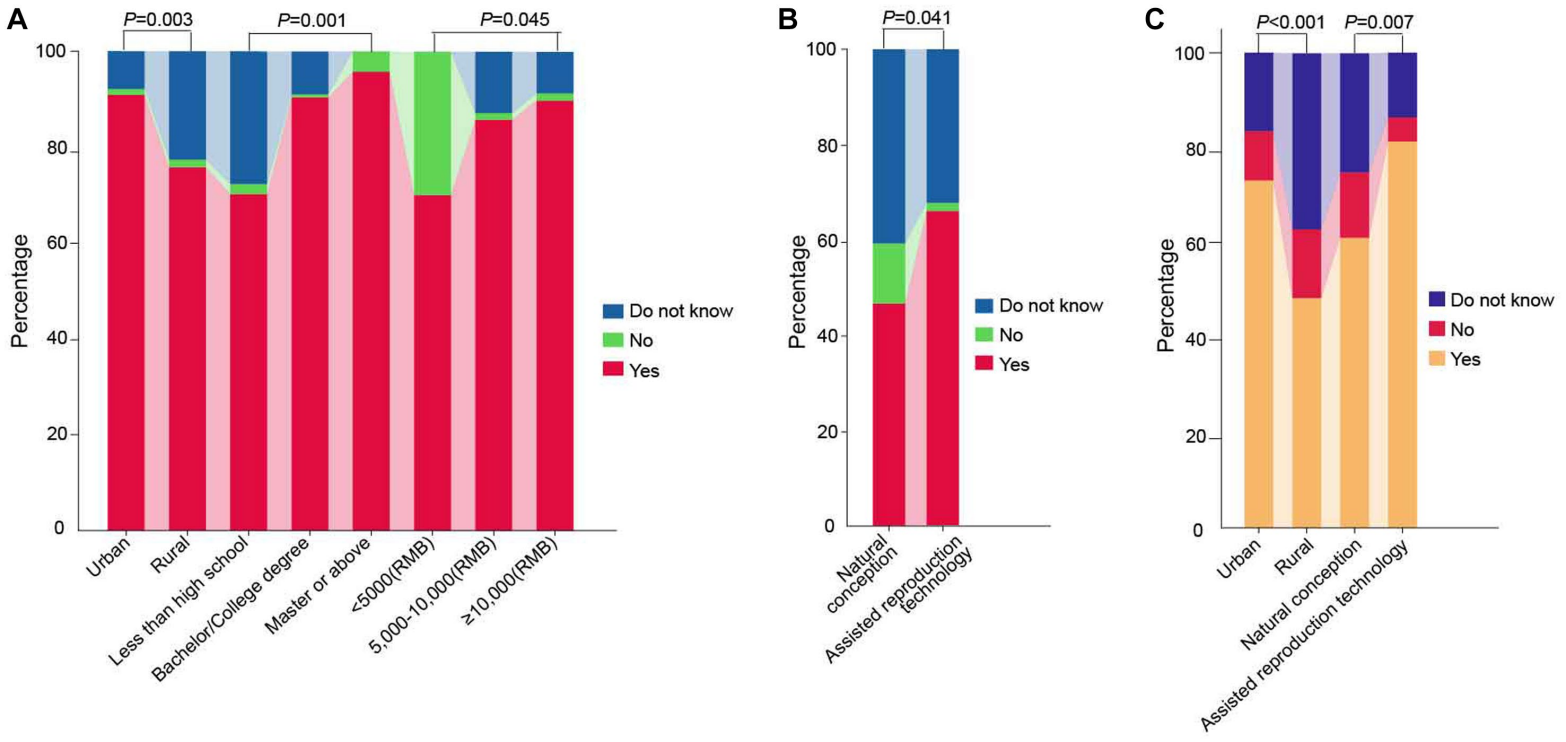
Comparison of pregnant women’s perception and willingness of NIPT test results among different subgroups. **(A)** Perception-based questions 1: Do you believe prenatal screening and diagnosis are the primary measures for decreasing the incidence of birth defects? **(B)** Perception-based questions 4: Do you believe NIPT is more accurate and diagnostic-like than serum screening for chromosomal diseases? **(C)** Willingness-related questions 2: Would you opt for NIPT as a primary method for fetal aneuploidy screening in your subsequent pregnancy? NIPT represents non-invasive prenatal testing.

The study found that 86.7% of women believe prenatal screening and diagnosis can reduce birth defects, and over 50% believe all pregnant women should undergo chromosome disease screening. 95.5% of pregnant women will choose the screening method their doctor recommends. 51.8% believe NIPT is more accurate and closer to diagnostic technology than serum screening. The percentage of women who chose “no” for all Women’s perception of NIPT was less than 10% (questions P1–P5 in Table 2). Significant differences were observed among different subgroups of the place of residence, educational level, and family income regarding the perception of prenatal screening and diagnosis as the primary means of reducing birth defects (Table 4, Figure 2A). Regarding pregnant women’s perception of NIPT being more accurate and closer to diagnostic technology than serum screening, the awareness was significantly higher among women who underwent assisted reproductive technology than those who conceived naturally (*p* = 0.041) (Table 4, Figure 2B).

**Table 4:** Comparisons of women’s perception and willingness to NIPT among subgroups.

Characteristics		P1	P2	P3	P4	P5	W1	W2
Age	<35 years	0.478	0.058	0.397	0.119	0.136	0.473	0.383
	≥35 years							
Place of residence	Urban	0.003	0.034	0.673	0.062	0.363	0.139	<0.001
	Rural							
Educational level	Less than high school	0.001	0.158	0.479	0.201	0.128	0.643	0.345
	Bachelor/College degree							
	Master or above							
Family income (per month)	<5,000 (RMB)	0.045	0.527	0.461	0.25	0.745	0.247	0.233
	5,000–10,000 (RMB)							
	≥10,000 (RMB)							
Parity	0	0.759	0.425	0.018	0.219	0.804	0.564	0.135
	≥1							
Previous miscarriage or termination of pregnancy	Yes	0.183	0.642	0.567	0.765	0.228	0.831	0.61
	No							
Causes of abortion^a^	Fetal defects	0.643	0.521	0.806	0.312	0.373	0.298	0.26
	Non-fetal defects							
Conception way	Natural conception	0.306	0.946	0.305	0.041	0.307	0.969	0.007
	Assisted reproduction technology							
Gestational weeks (week)	12^+0^–22^+6^	0.727	0.681	0.357	0.915	0.178	0.955	0.46
	23^+0^–30^+6^							

P represents perception-based questions, and W represents willingness-related questions; values in the tables were expressed as values of *p*; all chi-square tests, educational level, and family income except for the Kruskal-Wallis test.

a 132 participants had previous miscarriages or termination of pregnancy.

### Pregnant women’s willingness on NIPT

The study found that 83.2% of pregnant women who were identified as high-risk by serum screening would choose NIPT, and 66.4% of pregnant women would directly choose NIPT for prenatal aneuploidy screening in their subsequent pregnancy, indicating a strong willingness among pregnant women to undergo NIPT. Urban pregnant women and those who underwent assisted reproductive technology had a stronger willingness to directly use NIPT for screening than rural pregnant women and those who conceived naturally (*p* < 0.001; *p* = 0.007) (Table 4, Figure 2C).

## Discussion

Multiple screening methods are available for screening T21, T18, and T13, such as maternal serum screening (MSS), ultrasound, and NIPT (24). However, MSS and ultrasound screening are known to have lower accuracy and higher false-positive rates (25, 26). Hence, the current common strategy in most countries involves combining these three screening methods. A meta-analysis indicated that the detection rate for T21 was 96%, 87% for T18, and 77% for T13 in the general population (27). The high accuracy of NIPT holds significant value in reducing invasive prenatal testing (28, 29). Nonetheless, despite its accuracy, NIPT has inherent constraints. Comprehension regarding the false positive rate of NIPT need to be improved. Two comprehensive reviews outlined the false positive rate for T21 via NIPT to be around 0.1% (30, 31), which was subsequently updated to 0.04% by Gil et al. (32). We believe that the study’s outcome is significantly influenced by whether the participating expectant women had previously undergone conventional MSS. Furthermore, only 33% of false-positive results for autosomal trisomy can be attributed to biological or technical factors, necessitating further research into the reasons for false positives in NIPT (33). NIPT can detect approximately 85% of fetal chromosomal abnormalities in women identified as high risk during early pregnancy screening (34). However, NIPT misses approximately 30.0% of chromosomal abnormalities that are detectable by invasive prenatal diagnostic methods (35). Despite its high accuracy, it is vital to acknowledge that NIPT is not a flawless technology. Inherent limitations and challenges persist, necessitating concomitant usage with other testing methodologies and clinical assessments for a holistic determination of fetal health (2, 36–38). Notably, NIPT hinges on free placental DNA and not – as certain literature posits – on “cell-free fetal DNA (cffDNA)” (2). The nuances of NIPT results are not thoroughly comprehended; uncertain variations may be enclosed within NIPT findings, demanding physicians proficiently relay this to expectant mothers undergoing the test (37, 39). The genesis of this research was to understand expectant women’s knowledge about NIPT, thus informing further propagation of knowledge on NIPT.

The ten knowledge questions in this study were designed based on the International Society for Prenatal Diagnosis (ISPD) Rapid Response Statement and previous related surveys (18, 40). Knowledge questions 1–5 were basic knowledge questions about NIPT, while questions 6–10 were too complex or challenging for most women to answer regarding the applicability of NIPT to special risk groups. In this study, only 60.6% of women knew that NIPT only screens for chromosomal abnormalities and that diagnostic testing requires invasive procedures. Additionally, only 76.5% of women were cognizant of the possibility of receiving an uninformative NIPT test result. In contrast, Kou et al.’s survey reported that more than 90% of pregnant women were aware of the limitations mentioned above of NIPT (18). The apparent disparity in cognizance could potentially be attributed to the varied demographics of the expectant pregnant women incorporated within our survey. Our study incorporates a significant 27.4% of pregnant women from the rural demographic; contrarily, the preponderance of contributors in Kou et al.’s investigation are derived from an urban milieu. Simultaneously, while women engaged in Kou et al.’s study—those with positive screenings who consequently proceeded with NIPT—were availed counseling and a mid-trimester abnormality scan during the questionnaire’s completion phase, we targeted expectant pregnant women demonstrating a critical risk of prevalent fetal chromosomal aneuploidy predicated upon MSS outcomes prior to initiating NIPT.

The present investigation revealed a lower rate of accurate responses to knowledge queries concerning the suitability of NIPT for special risk groups, except for the item that NIPT is only suitable for detecting single-fetal pregnancies, which was correctly answered by 54.4% of the participants. This outcome may be attributed to the technical specifications released by the China National Health and Family Planning Commission in 2016, which underscored the applicability of NIPT solely for detecting single-fetal pregnancies (9). This is consistent with the literature, which suggests that pregnant women usually have more knowledge about the practical aspects of chromosomal abnormality than the limitations and accuracy of screening (41, 42).

The study findings reveal that urban pregnant women exhibited a significantly higher rate of correct answers to two NIPT knowledge questions (K2 and K6) than their rural counterparts. Similarly, pregnant women with higher levels of education demonstrated a significantly higher rate of correct answers to three knowledge questions related to NIPT compared to those who were uneducated. Furthermore, pregnant women from higher-income households exhibited a significantly higher rate of correct answers to two NIPT knowledge questions than those from lower-income households. These results suggest that pregnant women in urban areas with higher income and higher education possess a greater understanding of NIPT, consistent with previous research (43, 44). Similar results were obtained in Women’s Perception of NIPT, where urban, high-income, and highly educated pregnant women had a significantly higher proportion in considering prenatal screening and diagnosis as the primary measure to reduce the incidence of birth defects, as compared to rural, low-income, and uneducated pregnant women.

If serum screening results suggest a high risk of chromosomal abnormalities in the subsequent pregnancy, a significant proportion of pregnant women (83.2%) would opt for NIPT to detect fetal aneuploidy. Furthermore, a considerable percentage of pregnant women (66.4%) would select NIPT as the primary method for fetal aneuploidy screening in the subsequent pregnancy. Conversely, alternative studies have reported that only approximately 30.4% of pregnant women would directly choose NIPT for fetal aneuploidy detection (18, 45). This discrepancy may be attributed to the higher income level of the surveyed households, with 60.2% of families earning a monthly income of ≥10,000 RMB. Additionally, the cost of NIPT has decreased to 1,100 RMB per test due to its increased utilization in China. In light of the potential hazards of miscarriage, a considerable majority (66.4%) of expectant mothers would opt for NIPT as their primary choice for prenatal screening in subsequent pregnancies.

The ACMG and the National Society of Genetic Counselors (NSGC) recommend that NIPT counseling should be provided by a qualified prenatal care provider, a trained designee, or a genetic counselor (46, 47). The present investigation revealed that 95.5% of pregnant women believed that medical practitioners’ recommendations are essential in selecting screening methods. A comparable inquiry discovered that women exhibited women preferred pre- and post-NIPT counseling by a midwife (48). As online information can have varying degrees of accuracy, it is vital to establish an effective prenatal screening counseling platform that provides professional and efficient counseling services to pregnant women, enabling them to make the best choice. Additionally, reducing the anxiety caused by positive results and placing the patient at the center of decision-making are crucial factors in determining the uptake of diagnostic testing (49).

NIPS is classified as a screening test rather than a diagnostic test. To ensure consistency in testing methods and procedures, prominent governing and professional organizations, including the American College of Obstetricians and Gynecologists, ACMG, NSGC, and ISPD, recommend standardization of NIPT. These organizations also advise that informed decision-making, education, and counseling should be provided before and after testing (45–47, 50). At the national level of healthcare, the government must prioritize the reduction of superfluous invasive testing and miscarriage-related outcomes, as well as the formulation of more efficacious policies for prenatal care to mitigate the prevalence of birth defects. The findings of this study indicate that, despite the strong preference of Chinese expectant mothers for NIPT, their knowledge and perception of this screening method for fetal aneuploidy is limited. Consequently, it is incumbent upon the government to implement effective measures to enhance public awareness of fetal aneuploidy screening and promote the judicious use of NIPT.

This study is subject to certain limitations. Firstly, Given that our study exclusively focused on pregnant women residing in Zhejiang Province, China, it is important to note that the generalizability of our findings is limited not only to other countries but also to the wider Chinese population. Secondly, the study was conducted in developed urban areas of China, and the sample size was relatively small, necessitating larger sample sizes and surveys across multiple cities. Finally, while the study’s questionnaire was informed by prior research, it lacked expert validation, evaluation of questionnaire applicability, and reliability. The questionnaire will be further improved in future research.

## Conclusion

NIPS is a screening test that is distinct from a diagnostic test. Standardization of the testing methods and procedures utilized in NIPS is imperative. NIPT should be administered through informed decision-making, education, and counseling before and after testing. Chinese expectant mothers strongly prefer NIPT yet possess limited knowledge and comprehension of this screening method for fetal aneuploidy. Consequently, it is incumbent upon the government to implement effective measures to augment public awareness of fetal aneuploidy screening and promote appropriate utilization of NIPT.

## Data availability statement

The original contributions presented in the study are included in the article/supplementary material, further inquiries can be directed to the corresponding authors.

## Ethics statement

The studies involving humans were approved by written informed consent was taken from all participants. The study was approved by the Women’s Hospital, School of Medicine, Zhejiang University Ethics Committee (approval number: IRB-20210185-R), and obtained informed consent from each participant following the Helsinki Declaration. The studies were conducted in accordance with the local legislation and institutional requirements. The participants provided their written informed consent to participate in this study.

## Author contributions

YZ, ZX, YG, JZ, MH, and MJ made a significant contribution to the work reported, whether that is in the conception, study design, execution, acquisition of data, analysis and interpretation, or in all these areas, and took part in drafting, revising or critically reviewing the article. All authors contributed to the article and approved the submitted version.

## References

[1] Benn P Borell A Chiu R Cuckle H Dugoff L Faas B. Position statement from the aneuploidy screening committee on behalf of the Board of the International Society for Prenatal Diagnosis. Prenat Diagn. (2013) 33:622–9. doi: 10.1002/pd.4139 23616385

[2] Liehr T Harutyunyan T Williams H Weise A. Non-invasive prenatal testing in Germany. Diagnostics. (2022) 12:2816. doi: 10.3390/diagnostics12112816, PMID: 36428876 PMC9689121

[3] Wen L Gao J Huang L Li D Zhong G. Noninvasive prenatal screening in Southeast China: clinical application and accuracy evaluation. Expert Rev Mol Diagn. (2022) 22:841–8. doi: 10.1080/14737159.2022.2125803, PMID: 36107698

[4] Allyse M Minear MA Berson E Sridhar S Rote M Hung A. Non-invasive prenatal testing: a review of international implementation and challenges. Int J Women's Health. (2015) 7:113–26. doi: 10.2147/IJWH.S67124, PMID: 25653560 PMC4303457

[5] Xie X Wang M Goh ES Ungar WJ Little J Carroll JC. Noninvasive prenatal testing for Trisomies 21, 18, and 13, sex chromosome aneuploidies, and microdeletions in average-risk pregnancies: a cost-effectiveness analysis. J Obstet Gynaecol Can. (2020) 42:740–749.e12. doi: 10.1016/j.jogc.2019.12.007, PMID: 32008974

[6] Gregg AR Skotko BG Benkendorf JL Monaghan KG Bajaj K Best RG. Noninvasive prenatal screening for fetal aneuploidy, 2016 update: a position statement of the American College of Medical Genetics and Genomics. Genet Med. (2016) 18:1056–65. doi: 10.1038/gim.2016.97, PMID: 27467454

[7] National Health and Family Planning Commission of China. Technical specification for prenatal screening and diagnosis of fetal free DNA in maternal peripheral blood. (2016). Available at: http://www.nhc.gov.cn/cms-search/xxgk/getManuscriptXxgk.htm?id=0e6fe5bac1664ebda8bc28ad0ed68389

[8] UK National Screening Committee. Fetal anomaly screening programme handbook (2023). Available at: https://www.gov.uk/government/publications/fetal-anomaly-screening-programme-handbook/overview#governance

[9] China National Health and Family Planning Commission. The technical specifications of prenatal screening and diagnosis of fetal free DNA in peripheral blood of pregnant women (2016). Available at: http://www.nhc.gov.cn/cms-search/xxgk/getManuscriptXxgk.htm?id=0e6fe5bac1664ebda8bc28ad0ed68389

[10] Prenatal Screening and Diagnosis Expert Committee of China National Center for Clinical Laboratories. Consensus of laboratory technical experts for prenatal screening of fetal cell-free DNA in maternal peripheral blood. Chin J Lab Med. (2019) 42:341–6. doi: 10.3760/cma.j.issn.1009-9158.2019.05.005

[11] Inter-laboratory Quality Assessment of Prenatal Screening and Diagnostic Laboratories Expert Committee of China National Center for Clinical Laboratories. Expert consensus on quality evaluation indicators for prenatal screening. Chin J Med Genet. (2019) 36:413–8. doi: 10.3760/cma.j.issn.1003-9406.2019.05.001 31030423

[12] Juntao L. International guideline and national consensus of non-invasive prenatal testing. Chine J Pract Gynecol Obstet. (2017) 33:4. doi: 10.19538/j.fk2017060104

[13] Thomas SP Keller MA Ranson T Willard RE. Patient perspectives on noninvasive prenatal testing among black women in the United States: a scoping review. BMC Pregnancy Childbirth. (2023) 23:183. doi: 10.1186/s12884-023-05423-w, PMID: 36927679 PMC10018979

[14] Quaresima P Visconti F Greco E Venturella R Di Carlo C. Prenatal tests for chromosomal abnormalities detection (PTCAD): pregnant women's knowledge in an Italian population. Arch Gynecol Obstet. (2021) 303:1185–90. doi: 10.1007/s00404-020-05846-2, PMID: 33111167

[15] Kosec V Zec I Tislarić-Medenjak D Kuna K Simundić AM Lajtman-Krizaić M. Pregnant women's knowledge and attitudes to prenatal screening for fetal chromosomal abnormalities: Croatian multicentric survey. Coll Antropol. (2013) 37:483–9. PMID: 23940994

[16] Birko S Ravitsky V Dupras C Le Clerc-Blain J Lemoine M-E Affdal AO. The value of non-invasive prenatal testing: preferences of Canadian pregnant women, their partners, and health professionals regarding NIPT use and access. BMC Pregnancy Childbirth. (2019) 19:22. doi: 10.1186/s12884-018-2153-y, PMID: 30630440 PMC6327577

[17] Smith SK Cai A Wong M Sousa MS Peate M Welsh A. Improving women's knowledge about prenatal screening in the era of non-invasive prenatal testing for Down syndrome – development and acceptability of a low literacy decision aid. BMC Pregnancy Childbirth. (2018) 18:499. doi: 10.1186/s12884-018-2135-0, PMID: 30558569 PMC6296052

[18] Kou KO Poon CF Tse WC Mak SL Leung KY. Knowledge and future preference of Chinese women in a major public hospital in Hong Kong after undergoing non-invasive prenatal testing for positive aneuploidy screening: a questionnaire survey. BMC Pregnancy Childbirth. (2015) 15:199. doi: 10.1186/s12884-015-0636-7, PMID: 26330276 PMC4557816

[19] Buchanan A Sachs A Toler T Tsipis J. NIPT: current utilization and implications for the future of prenatal genetic counseling. Prenat Diagn. (2014) 34:850–7. doi: 10.1002/pd.4382, PMID: 24711206

[20] Schwab D. Construct validity in organizational behavior. Res Organ Behav. (1980) 2:3–43.

[21] Rummel RJ. Applied factor analysis. Evanston, IL: Northwestern University Press (1970).

[22] MacCallum RC Widaman KF Preacher KJ Hong S. Sample size in factor analysis: the role of model error. Multivar Behav Res. (2001) 36:611–37. doi: 10.1207/s15327906mbr3604_06, PMID: 26822184

[23] Zhao Y Zhang L Geng Y. Clinical drug trial participation: perspectives of pregnant women and their spouses. Patient Prefer Adherence. (2021) 15:2343–2352. doi: 10.2147/ppa.S328969 PMID: 34707349 PMC8542578

[24] Zhao G Dai P Wang C Liu L Zhao X Kong X. Clinical application of noninvasive prenatal testing for sex chromosome aneuploidies in Central China. Front Med (Lausanne). (2021) 8:672211. doi: 10.3389/fmed.2021.672211, PMID: 35155454 PMC8825788

[25] Alldred SK Takwoingi Y Guo B Pennant M Deeks JJ Neilson JP. First and second trimester serum tests with and without first trimester ultrasound tests for Down's syndrome screening. Cochrane Database Syst Rev. (2017) 2017:Cd012599. doi: 10.1002/14651858.Cd012599, PMID: 28295159 PMC6464364

[26] Kagan KO Sonek J Sroka A Abele H Wagner P Prodan N. False-positive rates in screening for Trisomies 18 and 13: a comparison between first-trimester combined screening and a cfDNA-based approach. Arch Gynecol Obstet. (2019) 299:431–7. doi: 10.1007/s00404-018-4983-2, PMID: 30519751

[27] Taylor-Phillips S Freeman K Geppert J Agbebiyi A Uthman OA Madan J. Accuracy of non-invasive prenatal testing using cell-free DNA for detection of Down, Edwards and Patau syndromes: a systematic review and meta-analysis. BMJ Open. (2016) 6:e010002. doi: 10.1136/bmjopen-2015-010002, PMID: 26781507 PMC4735304

[28] Driscoll DA Gross S. Clinical practice. Prenatal screening for aneuploidy. N Engl J Med. (2009) 360:2556–62. doi: 10.1056/NEJMcp0900134 19516035

[29] Mazza V Pati M Bertucci E Re C Ranzi A Percesepe A. Age-specific risk of fetal loss post second trimester amniocentesis: analysis of 5043 cases. Prenat Diagn. (2007) 27:180–3. doi: 10.1002/pd.1647, PMID: 17238217

[30] Kagan KO Sonek J Wagner P Hoopmann M. Principles of first trimester screening in the age of non-invasive prenatal diagnosis: screening for chromosomal abnormalities. Arch Gynecol Obstet. (2017) 296:645–51. doi: 10.1007/s00404-017-4459-9, PMID: 28702698

[31] Gil MM Quezada MS Revello R Akolekar R Nicolaides KH. Analysis of cell-free DNA in maternal blood in screening for fetal aneuploidies: updated meta-analysis. Ultrasound Obstet Gynecol. (2015) 45:249–66. doi: 10.1002/uog.14791, PMID: 25639627

[32] Gil MM Accurti V Santacruz B Plana MN Nicolaides KH. Analysis of cell-free DNA in maternal blood in screening for aneuploidies: updated meta-analysis. Ultrasound Obstet Gynecol. (2017) 50:302–14. doi: 10.1002/uog.17484, PMID: 28397325

[33] Hartwig TS Ambye L Sørensen S Jørgensen FS. Discordant non-invasive prenatal testing (NIPT) – a systematic review. Prenat Diagn. (2017) 37:527–39. doi: 10.1002/pd.5049, PMID: 28382695

[34] Maxwell S Dickinson JE Murch A O'Leary P. The potential impact of NIPT as a second-tier screen on the outcomes of high-risk pregnancies with rare chromosomal abnormalities. Aust N Z J Obstet Gynaecol. (2015) 55:420–6. doi: 10.1111/ajo.12385, PMID: 26286670

[35] Wang J Wang ZW Zhou Q Zhang B Yin T Yu B. Lower detectability of non-invasive prenatal testing compared to prenatal diagnosis in high-risk pregnant women. Ann Transl Med. (2019) 7:319. doi: 10.21037/atm.2019.06.70 31475189 PMC6694278

[36] Bowman-Smart H Gyngell C Mand C Amor DJ Delatycki MB Savulescu J. Non-invasive prenatal testing for "non-medical" traits: ensuring consistency in ethical decision-making. Am J Bioeth. (2023) 23:3–20. doi: 10.1080/15265161.2021.1996659, PMID: 34846986 PMC7614328

[37] Sagi-Dain L Singer A Petersen OB Lou S Vogel I. Trends in non-invasive prenatal screening and invasive testing in Denmark (2000-2019) and Israel (2011-2019). Front Med (Lausanne). (2021) 8:768997. doi: 10.3389/fmed.2021.768997, PMID: 34869484 PMC8635699

[38] Carbone L Cariati F Sarno L Conforti A Bagnulo F Strina I. Non-invasive prenatal testing: current perspectives and future challenges. Genes (Basel). (2020) 12:15. doi: 10.3390/genes12010015, PMID: 33374411 PMC7824607

[39] Oliveri S Ongaro G Cutica I Menicucci G Belperio D Spinella F. Decision-making process about prenatal genetic screening: how deeply do moms-to-be want to know from non-invasive prenatal testing? BMC Pregnancy Childbirth. (2023) 23:38. doi: 10.1186/s12884-022-05272-z, PMID: 36653738 PMC9845820

[40] Benn P Borrell A Cuckle H Dugoff L Gross S Johnson JA. Prenatal detection of Down syndrome using massively parallel sequencing (MPS): a rapid response statement from a committee on behalf of the Board of the International Society for prenatal diagnosis, 24 October 2011. Prenat Diagn. (2012) 32:1–2. doi: 10.1002/pd.2919, PMID: 22275335

[41] Bangsgaard L Tabor A. Do pregnant women and their partners make an informed choice about first trimester risk assessment for Down syndrome, and are they satisfied with the choice? Prenat Diagn. (2013) 33:146–52. doi: 10.1002/pd.4026, PMID: 23225252

[42] Favre R Moutel G Duchange N Vayssière C Kohler M Bouffet N. What about informed consent in first-trimester ultrasound screening for Down syndrome? Fetal Diagn Ther. (2008) 23:173–84. doi: 10.1159/000116738, PMID: 18417975

[43] Pruksanusak N Suwanrath C Kor-Anantakul O Prasartwanakit V Leetanaporn R Suntharasaj T. A survey of the knowledge and attitudes of pregnant Thai women towards Down syndrome screening. J Obstet Gynaecol Res. (2009) 35:876–81. doi: 10.1111/j.1447-0756.2009.01035.x, PMID: 20149035

[44] Dahl K Hvidman L Jørgensen FS Henriques C Olesen F Kjaergaard H. First-trimester down syndrome screening: pregnant women's knowledge. Ultrasound Obstet Gynecol. (2011) 38:145–51. doi: 10.1002/uog.8839 20878670

[45] Taylor JB Chock VY Hudgins L. NIPT in a clinical setting: an analysis of uptake in the first months of clinical availability. J Genet Couns. (2014) 23:72–8. doi: 10.1007/s10897-013-9609-z, PMID: 23723049

[46] Gregg AR Gross SJ Best RG Monaghan KG Bajaj K Skotko BG. ACMG statement on noninvasive prenatal screening for fetal aneuploidy. Genet Med. (2013) 15:395–8. doi: 10.1038/gim.2013.29, PMID: 23558255

[47] Devers PL Cronister A Ormond KE Facio F Brasington CK Flodman P. Noninvasive prenatal testing/noninvasive prenatal diagnosis: the position of the National Society of Genetic Counselors. J Genet Couns. (2013) 22:291–5. doi: 10.1007/s10897-012-9564-0, PMID: 23334531

[48] Lewis C Silcock C Chitty LS. Non-invasive prenatal testing for Down's syndrome: pregnant women's views and likely uptake. Public Health Genomics. (2013) 16:223–32. doi: 10.1159/000353523, PMID: 23886854

[49] Gidiri M McFarlane J Holding S Morgan RJ Lindow SW. Uptake of invasive testing following a positive triple test for Down's syndrome. Are midwives different counsellors compared with obstetricians? J Obstet Gynaecol. (2007) 27:148–9. doi: 10.1080/01443610601113946, PMID: 17454460

[50] Tian M Feng L Li J Zhang R. Focus on the frontier issue: progress in noninvasive prenatal screening for fetal trisomy from clinical perspectives. Crit Rev Clin Lab Sci. (2023) 60:248–69. doi: 10.1080/10408363.2022.2162843 36647189

